# Comparison of Artificial Intelligence and Radiologists in MRI-Based Prostate Cancer Diagnosis: A Meta-Analysis of Accuracy and Effectiveness

**DOI:** 10.3390/biomedicines14010020

**Published:** 2025-12-21

**Authors:** Huiqi Chen, Erwang Li, Paul J. Christos, Yuan-Shan Zhu

**Affiliations:** 1 Graduate Program of Population Health Sciences, Weill Cornell Medicine, Cornell University, New York, NY 10065, USA; erl4012@alumni.weill.cornell.edu (E.L.); pac2001@med.cornell.edu (P.J.C.); 2 Department of Population Health Sciences, Weill Cornell Medicine, New York, NY 10065, USA; 3 Graduate Program of Translational & Clinical Investigation, Weill Cornell Graduate School of Medical Sciences, Cornell University, New York, NY 10065, USA; 4 Department of Medicine, Clinical & Translational Science Center, Weill Cornell Medicine, New York, NY 10065, USA

**Keywords:** prostate cancer, MRI, machine learning, artificial intelligence, deep learning, radiologists, meta-analysis

## Abstract

**Background:** Prostate cancer remains a leading cause of mortality in men, making early, accurate detection crucial for early intervention. While radiologists utilize the Prostate Imaging Reporting and Data System (PI-RADS) for the interpretation of MRI imaging, variations in expertise and inter-reader differences can affect diagnostic accuracy. Artificial intelligence (AI) has emerged as a promising tool for automated detection, with the potential to achieve diagnostic performance comparable to radiologists in identifying clinically significant prostate cancer (csPCa), streamline workflows, and reduce unnecessary biopsies. However, its real-world performance compared to expert radiologists remains a topic of ongoing debate. **Purpose:** This meta-analysis aims to evaluate whether AI can achieve diagnostic performance that is comparable to that of radiologists in MRI-based prostate cancer detection by comparing diagnostic accuracy, sensitivity, specificity, and area under the receiver operating characteristic curve (AUROC). **Methods:** Following PRISMA 2020 guidelines, we searched PubMed for studies directly comparing AI and radiologists in MRI-based detection of csPCa. Ten studies (20,423 patients) were included, and quality was assessed using QUADAS-2. Analyses included forest plots for diagnostic sensitivity and specificity, funnel plots of AUROC to assess publication bias, and paired AUROC difference plots to directly compare diagnostic accuracy. **Results:** Pooled sensitivity was 0.87 (95% CI: 0.81–0.94) for AI and 0.85 (95% CI: 0.77–0.94) for radiologists; pooled specificity was 0.61 (95% CI: 0.51–0.72) for AI and 0.63 (95% CI: 0.54–0.71) for radiologists. Funnel plots of AUROC against standard error showed no strong visual evidence of publication bias. Paired AUROC difference analysis demonstrated no significant performance difference between AI and radiologists, with a pooled difference of 0.018 (*p* = 0.378). **Conclusions:** AI systems demonstrated diagnostic performance comparable to radiologists for MRI-based detection of csPCa, with a nonsignificant and slightly higher pooled sensitivity and AUROC. Moreover, AI has the potential to improve workflow speed, uniformity across expertise levels, and hybrid AI-radiologist approaches to reduce unnecessary biopsies. Large-scale, prospective trials with standardized protocols are needed to assess AI’s effectiveness across diverse clinical settings.

## 1. Introduction

Prostate cancer is the most commonly diagnosed cancer in men in the United States in 2025, with an estimated 313,789 new cases. This makes up 30% of all male cancer diagnoses [1]. Prostate cancer prevalence is also notably high in Europe. The age-standardized incidence rate is 111.6 per 100,000 men in Northern Europe and 97.2 per 100,000 men in Western Europe [2]. Multiparametric magnetic resonance imaging, known as mpMRI, is crucial in the early detection of prostate cancer and in the reduction of unnecessary biopsies, as it helps doctors decide the need and the significance of a biopsy [3]. Radiologists use the Prostate Imaging Reporting and Data System (PI-RADS), a structured approach, to interpret prostate MRI scans. However, despite this standardized system, the accuracy of interpretations still varies. Studies show only moderate to fair agreement between readers, even in organized screening settings [4].

Artificial intelligence (AI), particularly deep learning models, is being explored to improve the interpretation of prostate MRI images. By using deep learning reconstruction techniques, AI systems have demonstrated a significant potential for improving image quality, improving efficiency, and patient experiences when compared to conventional imaging techniques [5]. These tools also shorten scan time and may help streamline radiology workflows [3,5]. In the field of prostate cancer detection, AI models are playing an increasingly important role. They assist in detecting lesions, classifying and segmenting them, as well as in PI-RADS scoring. In this way, these applications can support more consistent interpretation across different experience levels [3]. One of AI’s key advantages is its ability to reduce radiologist variability—a long-standing problem in prostate MRI interpretation. AI may also help standardize reporting and strengthen quality control in structured screening pathways [4].

Even though AI holds promise for prostate cancer imaging, there has not been a meta-analysis that systematically assesses its performance compared to radiologists using MRI. Current studies are often different in design, reporting standards, datasets, and diagnostic metrics, which makes direct comparison difficult [4]. Previous systematic reviews have described similar challenges but were limited by small, internally validated datasets [6,7]. Many relied on narrative summaries because of high heterogeneity and inconsistent reporting. As a result, they were unable to provide clear quantitative evidence comparing AI with radiologists across diverse clinical settings [6,7]. Assessment in various clinical contexts is made more difficult by reader experience variability and a lack of standardization in interpretation [4]. Without consolidated evidence, it remains unclear whether AI can consistently match radiologists or if it should be used primarily as a diagnostic support tool. Therefore, a meta-analysis is needed to synthesize the available data and provide clarity on AI’s role in MRI-based prostate cancer detection.

This meta-analysis aims to evaluate the diagnostic performance of AI models directly compared to radiologists in MRI-based prostate cancer detection. Specifically, it assesses sensitivity, specificity, and diagnostic accuracy across multiple studies to determine whether AI can reliably support or enhance human interpretation. This study aims to shed light on the advantages and disadvantages of AI in order to help radiologists who are looking for tools to support decision-making, researchers who are creating diagnostic algorithms, and imaging and pharmaceutical companies that are looking for scalable, clinically validated technology. It also addresses the broader requirement for standardization in prostate MRI interpretation and quality assurance, which is essential for integrating AI in real-world workflows [4].

## 2. Methods

### 2.1. Search Strategy

This meta-analysis adhered to the reporting specifications outlined in the PRISMA 2020 guidelines [8]. A systematic literature search was conducted using the PubMed database to identify eligible studies directly comparing AI with radiologists in MRI-based prostate cancer diagnosis. A comprehensive literature search was conducted in PubMed from database inception to 14 March 2025, with the search scope limited to English-language human research. The search strategy followed a structured approach using Medical Subject Headings (MeSH) and keyword combinations to maximize coverage. Key concepts and their variations included: “Artificial Intelligence” or “Machine Learning” or “algorithm”, “Prostatic Neoplasms” or “Prostate Cancer”, “Magnetic Resonance Imaging”, “Radiologists”. Four structured queries were developed to capture relevant studies. The first three queries were executed as keyword-based searches in PubMed, using the following terms: (1) “Artificial intelligence in prostate cancer diagnosis,” (2) “(Artificial intelligence OR Machine Learning OR algorithm) in prostate cancer diagnosis,” (3) “(Artificial intelligence OR Machine Learning OR algorithm) in MRI prostate cancer diagnosis”. The fourth query used a MeSH-guided formulation to ensure comprehensive coverage of indexed terms: (4) (“Artificial Intelligence” [MeSH] OR “Machine Learning” [MeSH]) AND (“Prostatic Neoplasms” [MeSH] OR “Prostate Cancer” [tiab]) AND “Magnetic Resonance Imaging” [MeSH] AND “Radiologists” [tiab])”. To ensure inclusion of studies with direct clinical comparison, PubMed Publication Type filters were applied during screening: AND (Clinical Study [pt] OR Comparative Study [pt] OR Randomized Controlled Trial [pt]). This ensured that diagnostic studies comparing AI models with radiologists in prostate MRI were included, while non-comparative or algorithm development studies were excluded at the eligibility stage. Additional manual screening of reference lists from relevant reviews and included studies was conducted to ensure comprehensive coverage. The references of included studies were manually reviewed to identify additional eligible citations. Because this meta-analysis involved only publicly available, previously published data, no institutional review board approval or patient consent was required. The systematic review has been registered with INPLASY (INPLASY Registration Code: INPLASY2025110042).

### 2.2. Eligibility Criteria

Eligible studies met the following criteria: (1) Participants had to be adult patients (age ≥ 18 years) undergoing MRI-based evaluation for suspected or confirmed prostate cancer. (2) The study population consisted of individuals undergoing diagnostic assessment, with most studies focusing on the detection of csPCa. (3) Only clinical diagnostic studies were included. These could be retrospective, comparative, prospective, or randomized control in design. (4) Each study had to directly compare the performance of artificial intelligence (AI)-based models with that of radiologists in interpreting prostate MRI. (5) Studies were required to report at least one diagnostic accuracy metric, including sensitivity, specificity, or area under the receiver operating characteristic curve (AUC). Time efficiency or cost-effectiveness outcomes were noted if available. (6) A reported patient sample size was required, with preference given to studies including ≥50 participants. (7) Only full-text peer-reviewed articles published in English were included. (8) Studies had to focus strictly on the diagnostic phase of prostate cancer evaluation; those testing AI-guided biopsy or procedural interventions were excluded.

Studies were excluded based on the following criteria: (1) Research focusing on AI model development or algorithmic validation without clinical application or real-world testing. (2) Studies without a direct comparison between AI models and radiologists. (3) Research using non-MRI imaging modalities, such as ultrasound, computed tomography (CT), or positron emission tomography (PET). (4) Case reports, editorials, conference abstracts, and narrative or systematic reviews were excluded unless they contained original aggregated data meeting inclusion standards. (5) Studies that lacked complete diagnostic metrics or failed to report sample size. (6) Preclinical or animal studies were not considered. (7) Studies involving AI-guided biopsies, robotic interventions, or post-diagnostic treatment assistance were excluded unless the primary aim was to assess diagnostic accuracy during initial MRI evaluation. In cases of duplicate or overlapping datasets, the most recent and comprehensive publication was selected.

### 2.3. Data Extraction

Data were extracted independently by the lead author using a structured table developed to capture both methodological consistency and key performance metrics across studies. The extracted variables included study title, study type (prospective, retrospective, comparative), authorship, year of publication, reference standard, number of MRI test cases and patients, AI model architecture, and radiologist interpretation method. A comprehensive overview of baseline characteristics is summarized in Table 1. Although ten studies satisfied the inclusion criteria for this meta-analysis, subgroup-level data were extracted and analyzed separately when applicable, such as in studies that used different internal and external test sets, multicenter datasets, or radiologist-specific sub-cohorts. This strategy was used to maintain methodological transparency and better account for within-study variation in validation design and diagnostic performance.

Primary outcomes of interest were sensitivity and specificity for both artificial intelligence (AI) systems and radiologists, reported with corresponding 95% confidence intervals (CIs) and standard errors (SEs), where available. Data were extracted at the lesion level when reported, except for Giganti et al. [12] and Bayerl et al. [13], which presented only patient-level performance metrics. Sensitivity was evaluated based on a clinical threshold of Gleason Grade Group (GGG) ≥ 2 and a radiological threshold of PI-RADS ≥ 3. Notable outliers were Zhao et al., who defined clinical significance more broadly as GGG ≥ 1 (i.e., Gleason score ≥ 3 + 3), and Saha et al., who also used a PI-RADS ≥ 3 threshold but adhered to the GGG ≥ 2 criterion. The area under the receiver operating characteristic curve (AUROC), along with associated CIs or SEs, was also extracted as a key summary measure of diagnostic accuracy.

Study screening and data extraction were carried out by the first author (H.C.), and all extracted values were independently reviewed by the second author (E.L.). Any differences in study eligibility or extracted data were resolved through discussion, with final agreement reached in consultation with the senior author (Y.-S.Z.). No automation tools or other machine-assisted screening software were used during identification, screening, or data extraction.

### 2.4. Data Quality Assessment

To assess the methodological quality and risk of bias of the included studies, we used the Quality Assessment of Diagnostic Accuracy Studies-2 (QUADAS-2) tool, as recommended for diagnostic accuracy reviews [19]. This tool evaluates the risk of bias and applicability across four domains: (1) patient selection, (2) index test, (3) reference standard, and (4) flow and timing.

Publication bias was assessed using funnel plots of study-level AUROC against its standard error. Standard errors were either extracted or approximated from reported 95% CIs. A random-effects reference line from DerSimonian–Laird τ^2^ [20] with Hartung–Knapp adjustment [21] was added. Small-study effects were then evaluated with Egger’s regression test. Funnel plots were generated in R version 4.5.1 using the meta package (version 8.2-1).

### 2.5. Statistical Analysis

First, the pooled diagnostic sensitivity and specificity were calculated using a DerSimonian–Laird random effects model. To address the limitations of this method in the presence of high heterogeneity and to obtain more robust error estimates, the Hartung-Knapp (HK) adjustment was applied to calculate the 95% confidence intervals. The 95% confidence intervals were obtained using the Wald normal approximation method [20]. The heterogeneity was evaluated using Cochran’s Q test, and an *I*^2^ value greater than 50% was considered substantial heterogeneity [22].

Next, the paired difference in AUROC between AI and radiologists was calculated by subtracting the value of radiologists from the value of the AI in the same study (AI—Radiologist) [23]. These paired differences were synthesized using an inverse-variance random-effects meta-analysis based on the DerSimonian–Laird estimator with Hartung–Knapp adjustment. The corresponding standard errors were derived from the reported or back-calculated values.

For AUROC and specificity, all studies were included. For sensitivity, however, studies that reported a standard error of zero (SE = 0) were excluded because these values usually result from rounding very small SEs. This exclusion prevented singular weights and avoided infinite weighting in the meta-analysis model [24]. All statistical analyses were performed using R (version 4.5.1) with the meta (version 8.2-1), dplyr (version 1.1.4), and grid (base, R 4.5.1) packages. A *p* < 0.05 was considered statistically significant.

## 3. Results

### 3.1. Study Selection

**Figure 1:**
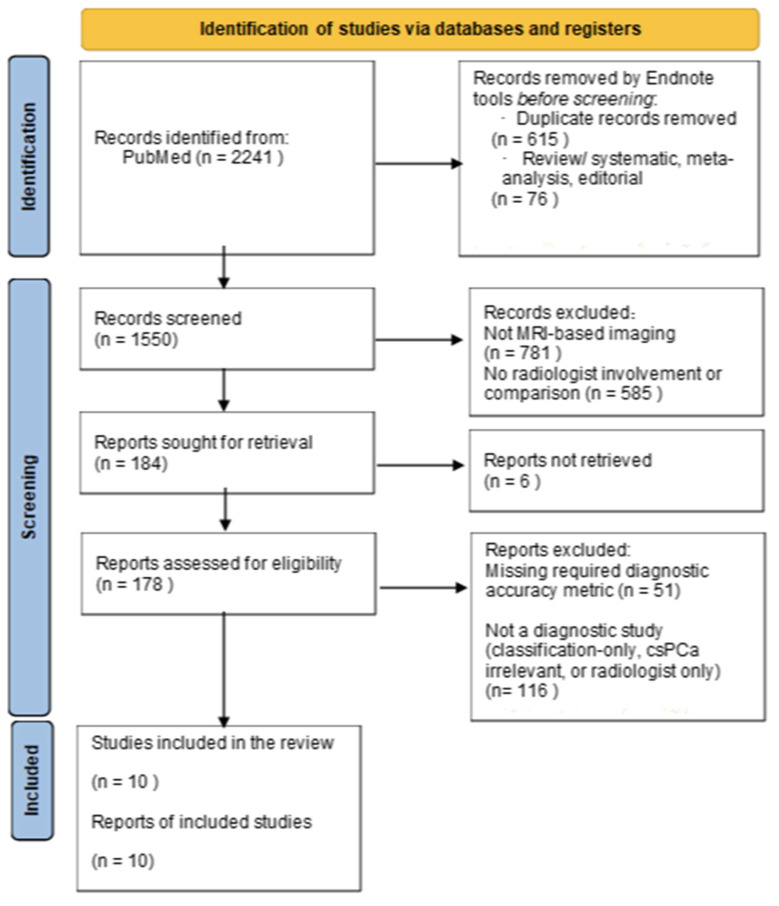
Literature Reviews and Study Selection flowchart.

The initial search generated 2241 articles, and 10 eligible studies were included, as illustrated in the PRISMA flow diagram (Figure 1). All studies and reports were retrospective and provided a direct comparison of MRI interpretation between radiologists and various AI models. For all the studies, the radiologists used the PI-RADS (v2.1) for the interpretation of MRI imaging, whereas a different AI model was used for each of the 10 studies (Table 1). Out of the ten studies, five used 2 or 3 different databases for this direct comparison. The characteristics of these studies are summarized in Table 1.

### 3.2. Quality Assessment

**Figure 2:**
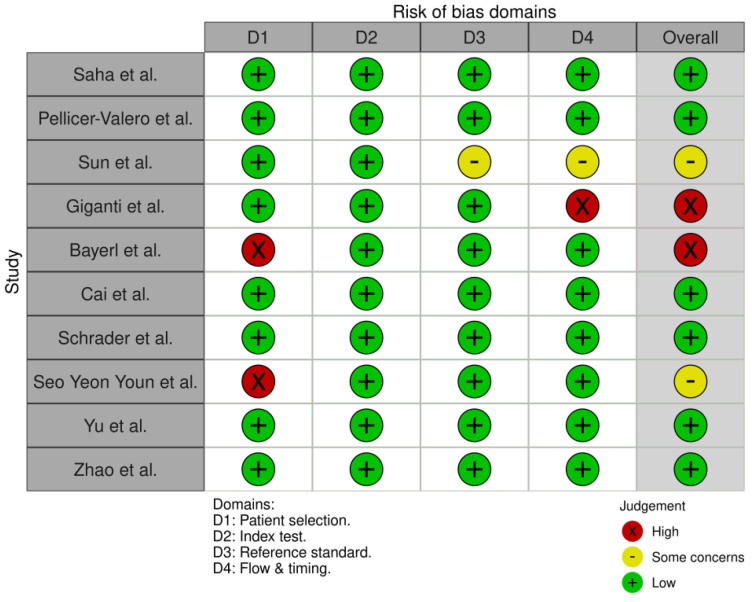
Risk of Bias Summary.

A visual summary of the risk-of-bias assessment is presented in Figure 2, generated using the ROBVIS tool [19]. Overall, 7 out of 10 studies (70%) were rated as having low risk of bias in all domains. Three studies had domain-specific concerns. Giganti’s study had a substantial risk in flow and timing. Nearly half of the patients (46%) were unbiopsied and presumed negative, which introduced verification bias. Bayerl’s [13] study had a high risk of bias in patient selection due to its retrospective design and lack of direct radiologist comparison. Seo Yeon Youn’s [16] study also showed a high risk in patient selection due to exclusion criteria. However, it maintained low risk in all other domains, resulting in moderate overall bias. Three studies showed domain-specific concerns, primarily related to retrospective design or incomplete verification. The internal validity of comparisons was supported by the majority of research, which used strong reference standards and tested AI and radiologists on the same datasets. No studies were excluded based on risk of bias.

### 3.3. Publication Bias Analysis

To evaluate potential publication bias, funnel plots of AUROC against the standard error were constructed for both radiologist and AI analyses. A symmetrical inverted funnel indicates the absence of publication bias, whereas asymmetry suggests potential small-study effects [25]. Egger’s regression test was used to support the visual assessment. The analyses and plots were generated in R version 4.5.1 using the meta (version 8.2-1) package.

**Figure 3:**
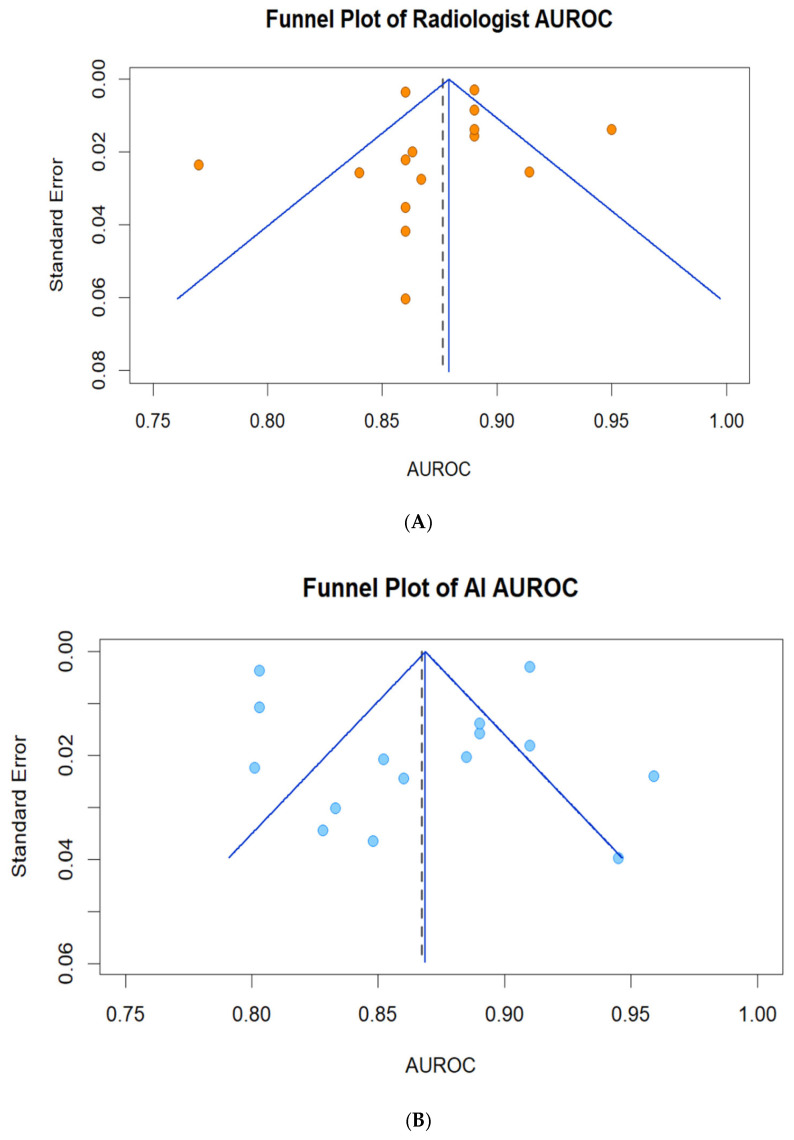
Funnel Plot of AUROC for Radiologists (**A**) and AI Models (**B**).

As shown in Figure 3A, the funnel plot for radiologists demonstrates a symmetrical distribution around the pooled AUROC (0.88), with only a slight asymmetry on the lower-AUROC side, where several higher-SE studies reported AUROCs below 0.85. For AI models (Figure 3B), the funnel plot exhibits a wider dispersion, including a few studies in the lower-left region (AUROC < 0.83), while lower-SE studies were more tightly clustered near the pooled value.

### 3.4. Overview of Diagnostic Sensitivity and Specificity

**Figure 4:**
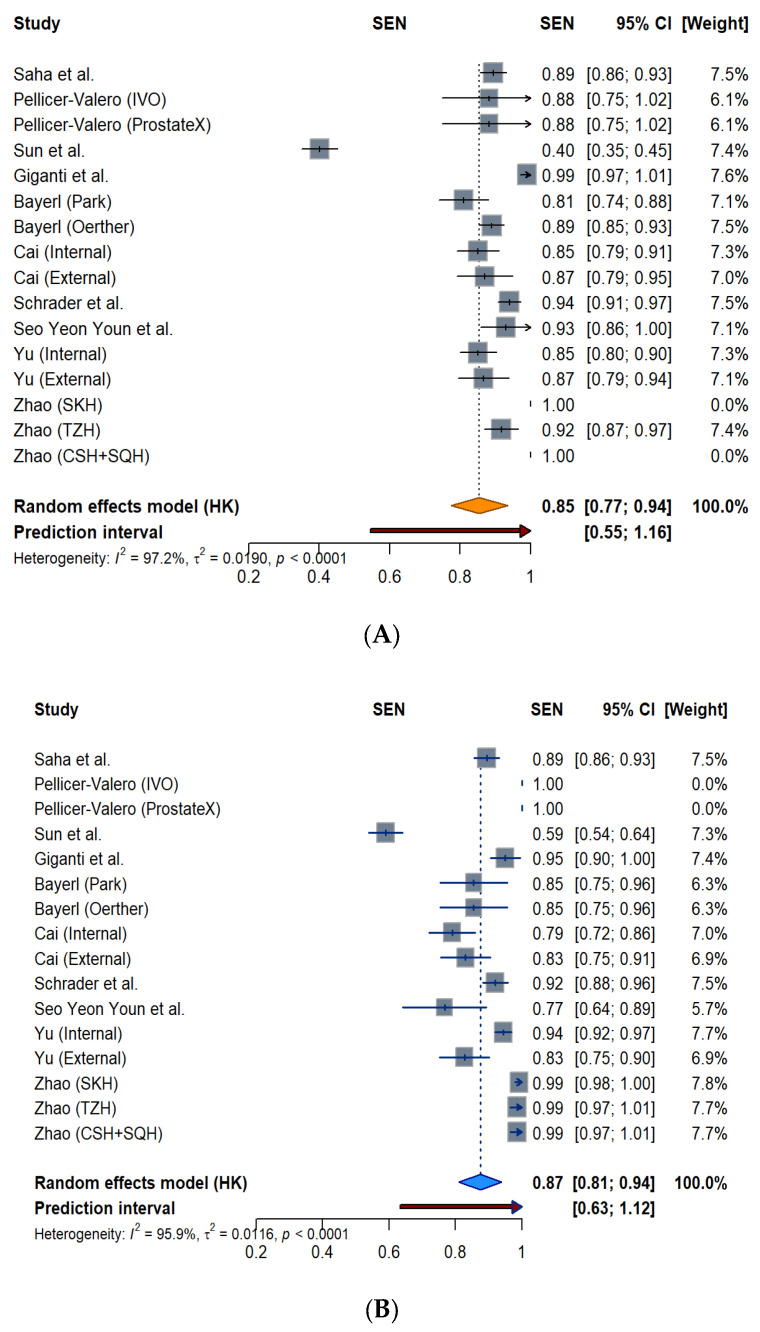
Forest plot of sensitivity estimates for radiologists (**A**) and AI models (**B**) across included studies.

To evaluate the diagnostic sensitivity of AI systems and radiologists in clinical settings, two forest plots were constructed [24]. Figure 4A presents the sensitivity estimates and corresponding 95% CIs for radiologists across individual studies or subgroups, while Figure 4B displays the equivalent data for AI models.

The pooled sensitivity for the radiologists was 0.85 (95% CI: 0.77–0.94) under a random-effects model, with substantial heterogeneity observed (*I*^2^ = 97.2%, *p* < 0.0001). The 95% prediction interval ranged from 0.55 to 1.00. Radiologist sensitivity values clustered between 0.81 and 0.89, while ranging from 0.40 to 0.99. By comparison, the pooled sensitivity of AI models (Figure 4B) was slightly higher at 0.87 (95% CI: 0.81–0.94) based on the random-effects model, with similarly high heterogeneity (*I*^2^ = 95.9%, *p* < 0.0001) and a 95% prediction interval of 0.63 to 1.00. Most AI estimates clustered between 0.83 and 0.94, while ranging from 0.59 to 1.0. Notably, AI models reached a perfect sensitivity (1.00) in Pellicer-Valero’s report. The AI sensitivity reached 0.99 for all three database analyses in Zhao’s report. On the other hand, Sun et al. reported a sensitivity of 0.59 using the AI model.

**Figure 5:**
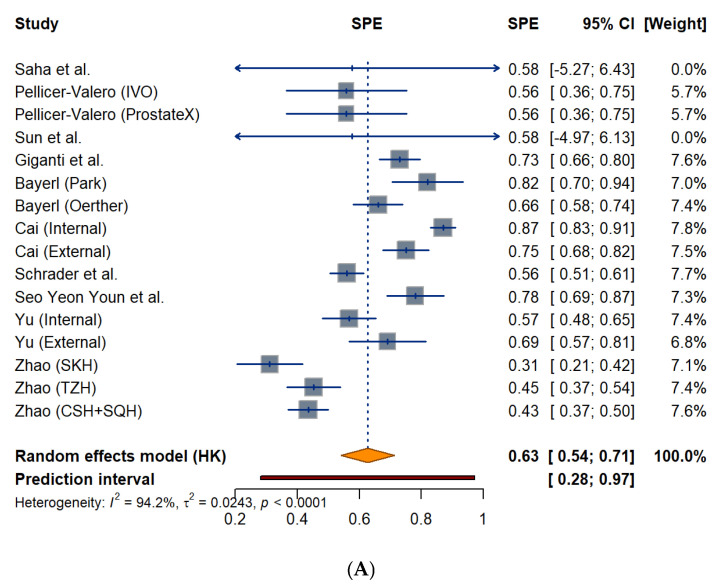

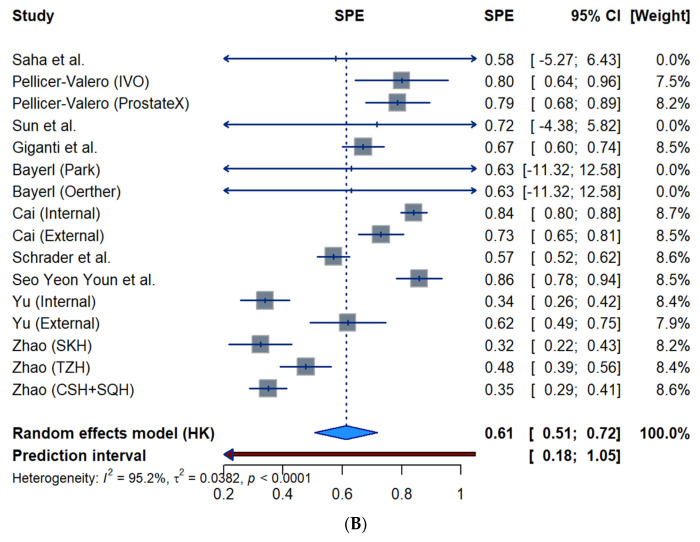
Forest Plot of Specificity Estimates for Radiologists (**A**) and AI models (**B**) Across Included Studies.

The pooled specificity for radiologists was 0.63 (95% CI: 0.54–0.71) under a random-effects model, with considerable heterogeneity (*I*^2^ = 94.2%, *p* < 0.0001). The calculated 95% prediction interval was wide, ranging from 0.28 to 0.97, as shown in Figure 5A forest plots. Radiologist-specificity estimates are most clustered between 0.56 and 0.87, with a range from 0.31 to 0.87. The pooled specificity for AI models (Figure 5B) was 0.61 (95% CI: 0.51–0.72) with similarly high heterogeneity (*I*^2^ = 95.2%, *p* < 0.0001). The 95% prediction interval is 0.18 to 1.00. Most AI estimates are between 0.57 and 0.86, with a range from 0.34 to 0.86.

To assess the robustness of these findings and potential bias arising from data granularity, a sensitivity analysis was performed by excluding 2 studies that utilized patient-level data (Giganti et al. [12] and Bayerl et al. [13]). After exclusion, the pooled sensitivity was 0.84 (95% CI: 0.74–0.95) for radiologists and remained at 0.87 (95% CI: 0.79–0.95) for AI models. For specificity, the pooled estimates adjusted to 0.61 (95% CI: 0.48–0.73) for both radiologists and AI systems. These values were closely aligned with the primary analysis, indicating that the inclusion of patient-level data did not substantially alter the overall diagnostic performance estimates. However, substantial heterogeneity persisted in this subgroup, suggesting that data granularity was not the primary cause of the observed variability.

### 3.5. AUROC Paired Difference Analysis

**Figure 6:**
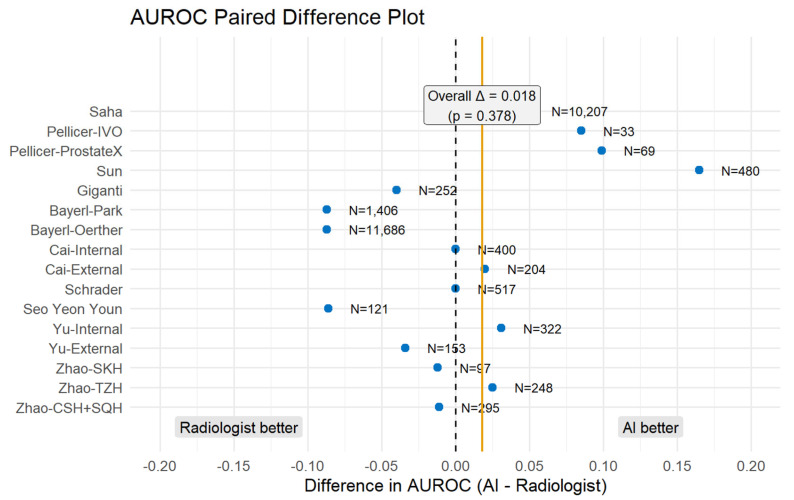
Paired Difference in AUROC Between AI and Radiologists. The dotted line indicates the baseline, and the solid yellow line indicates the overall change.

To further evaluate relative diagnostic performance, we compared the AUROC values of AI models with those of radiologists using a paired difference approach (Figure 6). This method directly quantifies whether AI or radiologists performed better within the same dataset by subtracting the radiologist AUROC from the AI AUROC [23]. Figure 6 shows that the majority of studies cluster around the baseline with an overall difference of 0.018 (*p* = 0.378), obtained from a random effects inverse-variance meta-analysis (DerSimonian–Laird + Hartung–Knapp). A positive value on the right side indicates an AI advantage, such as Saha (+0.05), Pellicer-Valero (ProstateX) (+0.099), Pellicer-Valero (IVO) (+0.085), and Sun (+0.16). On the other hand, a negative value on the left side indicates a radiologist’s advantage, such as Bayerl-Park, Bayerl-Oerther, and Seo Yeon Youn.

In the subgroup analysis excluding the research that used patient-level data (Giganti et al. [12] and Bayerl et al. [13]), the difference in AUROC between AI and radiologists increased slightly to 0.037 but remained statistically nonsignificant (*p* = 0.094). This finding further supports that AI performance is comparable to that of radiologists regardless of data granularity.

## 4. Discussion

### 4.1. Principal Findings and Clinical Implications

This meta-analysis integrated evidence from ten studies [9,10,11,12,13,14,15,16,17,18] comparing AI models and radiologists in MRI-based detection of csPCa. Across pooled data, AI models demonstrated sensitivity and accuracy comparable to or slightly better than those of radiologists, with similar specificity and largely overlapping confidence intervals. These data indicate that AI has the potential to improve prostate MRI interpretation without compromising diagnostic accuracy. Several studies also showed that AI can enhance workflow efficiency, assist less experienced readers, and balance detection and overestimation [16,26,27]. Together, these advantages relate directly to the objectives of increasing detection rates, lowering costs, and minimizing unnecessary interventions.

Previous systematic reviews in this field have faced several methodological shortcomings. Syer and colleagues [6] noted that “due to substantial heterogeneities in the included studies, a narrative synthesis is presented”, and concluded that there was insufficient evidence to suggest the clinical deployment of artificial intelligence algorithms at present. Their conclusions were primarily based on small, internally validated cohorts, with limited confirmation in external datasets. Similarly, Roest et al. [7] limited their analysis to eight studies with 7337 patients and focused exclusively on deep learning systems. They observed that the ongoing deep learning system had a lower sensitivity for PI-RADS ≥ 4 (84.2% vs. 88.8%, *p* = 0.43), and emphasized the continuing need for studies with larger datasets and for validation on external data. However, the present meta-analysis addresses these gaps by synthesizing evidence from 10 retrospective clinical diagnostic studies involving more than 20,000 patients, the largest and most comprehensive comparison to date. In contrast to previous reviews, our study evaluates sensitivity and specificity using random-effects pooling, and AUROC using paired head-to-head comparisons. This provides a more rigorous foundation for assessing the relative performance of radiologists and AI. Beyond the narrative or descriptive summary of previous reviews, our study offers a quantitative and clinically relevant benchmark by combining both radiomics-based and deep learning models, as well as by using pooled sensitivity, specificity, and paired AUROC difference analyses. This approach provides a clearer and more objective comparison of diagnostic performance. The findings suggest that AI performed slightly better or comparable to the radiologists in the interpretation of prostate MRI images.

One of the clearest advantages of AI is work efficiency and cost effectiveness. Evidence from individual multicenter trials suggests that AI may offer benefits in workflow efficiency and diagnostic confidence. In Sun et al.’s multicenter trial [26], AI-assisted MRI interpretation improved diagnostic confidence by 10.3% (*p* < 0.001) and reduced median reading time from 423 to 185 s, a 56.3% drop (*p* < 0.001). In another multicenter study [27] not included in this meta-analysis, the investigators reported similar findings, with reading time reduced by 351 s (*p* < 0.001) and confidence scores improved. Wang et al. [27] have suggested that handling high numbers of cases in a busy imaging center, this time reductions can contribute to faster report turnaround, less fatigue for radiologists, and even lower operational cost. A rigorous meta-analysis of reading time or diagnostic confidence was not possible because workflow-related outcomes were reported using disparate criteria and were not consistently assessed across studies. Therefore, rather than being definitive pooled estimates, the indicated workflow benefits should be considered preliminary and hypothesis-generating.

AI also demonstrated value in reducing variability in diagnostic performance across radiologists with different experience levels. In Youn’s [16] study, there were five groups of radiologists with different experiences who participated in the study: two resident groups (groups 1 and 2), two less-experienced radiologist groups (groups 3 and 4), and one expert subspecialist group (group 5). The AI system had an AUROC of 0.828, which was significantly higher than that of the resident reader (groups 1 and 2, AUROC 0.706; *p* = 0.011), comparable to less-experienced radiologists (groups 3 and 4), and lower than the expert subspecialist (group 5, AUROC 0.914; *p* = 0.013). This finding aligns with the negative paired difference observed for this study in our analysis (Figure 6), indicating a performance advantage for the expert radiologist over the standalone AI model. Similarly, Sun et al. [26] found that AI assistance significantly improved diagnostic performance among less-experienced radiologists in a two-center study, increasing lesion-level sensitivity from 0.78 to 0.88 and patient-level AUC from 0.84 to 0.89. This improvement corresponds to the positive difference (AI advantage) seen in our paired analysis, reflecting an AI-associated performance gain. Taken together, these findings suggest that AI may help improve diagnostic accuracy in prostate MRI interpretation. They also indicate that AI has the potential to reduce inter-reader variability in the diagnosis of csPCa.

Another important advantage of AI lies in its potential to reduce overestimation and unnecessary interventions through a hybrid diagnostic model. Cai et al. [14] demonstrated in the internal test set that AUROC increased from 0.89 (95% CI: 0.85–0.93) for image-only AI and 0.89 (95% CI: 0.86–0.93) for radiologists alone to 0.94 (95% CI: 0.91–0.96) when AI outputs were combined with radiologist interpretation (*p* < 0.001). Among pathology-proven cases, the hybrid approach achieved an AUROC of 0.87 (95% CI: 0.82–0.92) compared with 0.85 (95% CI: 0.80–0.90) for AI alone and 0.78 (95% CI: 0.72–0.83) for radiologists alone (*p* < 0.001). Consistent improvements were also observed in the external test set, with AUROC improving from 0.86 (95% CI: 0.80–0.91) for AI alone and 0.84 (95% CI: 0.79–0.90) for radiologists alone to 0.89 (95% CI: 0.84–0.93) with the hybrid model (*p* < 0.001). This data indicates the AI’s ability to characterize lesions reliably, which may avoid unnecessary biopsies and reduce health costs. However, hybrid AI–radiologist workflows varied substantially across studies. Before such approaches can be directly compared or widely used in clinical practice, more defined criteria and evaluation frameworks will be required.

When comparing across individual AI models, relative strengths differed by study design and outcome. Pellicer-Valero et al. [10] reported the highest AUROC (0.959) on the ProstateX dataset, with perfect sensitivity (1.00) and solid specificity (0.786). These results placed the algorithm at the top of the funnel plot and suggest excellent technical accuracy. However, this conclusion was obtained from a small, publicly accessible dataset, which raises questions regarding possible overfitting and its generalizability. Regarding Simon and Aliferis, “*the variance is a function of the learner and the sample size… low-bias models have higher variance, hence are unstable in small samples*.” [28]. This implies that complex AI models trained on small datasets often learn patterns that are specific to the development cohort. These patterns may not generalize well to new populations. Consequently, such models may perform well during internal validation, yet struggle to generalize reliably when applied to external cohorts.

In contrast, Saha et al. [9] used the biggest available cohort (N = 10,207) to provide the strongest evidence of reproducibility. With an AUROC of 0.91 and a sensitivity of 0.894, their AI system continuously outperformed radiologists in important performance parameters, contributing to the overall positive trend for AI. Conversely, studies such as Bayerl et al. [13] appeared on the negative side of the paired analysis, indicating instances where clinical radiologists outperformed the specific AI models tested. The relatively large weight of this study in pooled analysis highlights the importance of external validity and supports the stability of its findings. In addition, Cai et al. [14] also provided a different viewpoint. They showed that hybrid diagnostic approaches, which combine AI results with radiologists’ interpretation, greatly improved performance over either approach alone. These findings provide potential clues on how to use AI in the diagnosis of csPCa based on prostate MRI images.

### 4.2. Limitations

This meta-analysis also has several limitations. First, substantial heterogeneity (*I*^2^ > 90%) was observed across studies, which limits the direct comparability of pooled effect sizes. This heterogeneity likely reflects methodological and clinical differences, including variation in MRI acquisition protocols, patient demographics, and the diagnostic thresholds applied. In particular, PI-RADS cutoffs were not applied consistently across studies (e.g., ≥3 vs. ≥4), which shifts the balance between sensitivity and specificity. Clinical reference standards also differed, with most studies defining csPCa as GGG ≥ 2 and a minority using GGG ≥ 1. Because these thresholds determine how true-positive and false-negative cases are classified, such differences in both radiological and pathological criteria directly influence diagnostic estimates and contribute to between-study heterogeneity [4].

Second, the analysis included mixed data types, combining lesion-level data with patient-level data from two studies (Giganti et al. [12] and Bayerl et al. [13]). Although this variation in data aggregation could introduce bias, our sensitivity analysis excluding patient-level studies yielded results highly consistent with the primary analysis, confirming the robustness of our findings. Importantly, significant heterogeneity (*I*^2^ > 96%) persisted even after these studies were excluded, suggesting that variability was driven more by clinical and methodological differences than by the unit of analysis itself.

Third, variations in AI training and validation datasets, as well as insufficient reporting of important performance indicators like AUROC, confidence intervals, and standard errors, limited the ability to conduct fully standardized comparisons. These inconsistencies reduced the precision of pooled estimates. In addition, model architecture appeared to influence performance patterns. Radiomics-based models depend on handcrafted features and are more sensitive to variation in MRI acquisition. On the other hand, deep learning models learn directly from image data and may generalize better across scanners [29]. At the same time, deep learning systems require larger training datasets and can overfit when tested on small external cohorts [28]. Overall, because each study used different AI architectures with its own preprocessing choices and design objectives, there was insufficient methodological overlap to support meaningful subgroup analyses by model type. Overall, these factors help explain the variation in performance across AI systems in the included studies and underscore the need for more standardized frameworks in future research.

Fourth, although funnel plot analysis did not reveal any clear visual signs of publication bias, slight asymmetry among smaller studies with high variability suggests that selective reporting cannot be entirely excluded. However, regarding the potential overfitting in these smaller datasets (e.g., ProstateX), we applied a random effects inverse-variance weighting strategy [24]. This approach ensured that smaller studies did not disproportionately influence the pooled estimates, thereby preserving the validity of the overall findings.

Fifth, most included studies were conducted in high-resource academic centers, which may limit the generalizability of findings to other clinical settings, particularly those with different patient demographics or resource constraints. Beyond differences in model architecture and dataset size, an important consideration is the broader clinical applicability of AI performance. These studies relied on specialized MRI protocols, subspecialty radiologists, and advanced imaging equipment. Such conditions may not reflect routine practice in community hospitals or resource-limited environments. Variations in scanner quality, acquisition consistency, radiologist expertise, and patient populations may have a significant impact on diagnostic performance. This raises the question of whether AI systems tested only in large academic centers will perform as well in everyday clinical practice. Moreover, all included studies were retrospective, which introduces unavoidable sources of bias and heterogeneity related to data collection, study design, model selection, and interpretation.

## 5. Conclusions

This meta-analysis shows that artificial intelligence across different model types can achieve diagnostic performance comparable to that of radiologists in MRI-based detection of csPCa, with similar pooled AUROC, sensitivity, and specificity. Overall, these findings suggest that AI performance is statistically non-inferior to radiologist interpretation, supporting its potential role as a supportive diagnostic tool within prostate MRI workflows.

Beyond diagnostic accuracy, several individual studies have reported that AI assistance can improve workflow efficiency, reduce reading time, and enhance performance among less experienced readers [16,26,27]. As these outcomes were defined in different ways and could not be quantitatively pooled in this meta-analysis, they should be interpreted as preliminary observations rather than as generalizable effects. Nevertheless, they highlight areas in which AI may offer complementary clinical value, and underscore the need for standardized prospective evaluation.

Given the limitations of the current evidence, future research should prioritize large, prospective, multicenter studies that use standardized MRI acquisition protocols and consistent PI-RADS-based definitions of csPCa. Beyond technical validation, it will also be important to assess AI performance among radiologists with different levels of experience, particularly in settings where specialized prostate MRI expertise is limited. Such studies could clarify whether AI primarily serves as a performance equalizer or as an enhancer of expert-level interpretation. In addition, research in lower-resource healthcare systems is needed to assess the feasibility and generalizability of AI deployment outside academic centers. Finally, to better assess the clinical utility of AI-assisted pathways in routine prostate cancer diagnosis, future prospective trials should include practical outcomes such as cost-effectiveness, reduction of unnecessary biopsies, and downstream therapeutic effects.

## Figures and Tables

**Table 1:** Baseline Characteristics of Included Studies.

Study	Reference Standard	AI Model	Radiologists’ Method	Case Number	Sensitivity (AI)	Sensitivity (Rad)	Specificity (AI)	Specificity (Rad)	AUROC (AI)	AUROC (Rad)
Saha et al. 2024 [9]	Biopsy/prostatectomy	Ensemble DL Models	PI-RADS v2.1	10,207	0.894	0.577	0.577	0.577	0.91	0.86
Pellicer-Valero et al. (IVO) 2022 [10]	Biopsy	3D Retina U-Net	PI-RADS v2.1	33	1	0.558	0.800	0.558	0.945	0.86
Pellicer-Valero et al. (ProstateX) 2022 [10]	Biopsy	3D Retina U-Net	PI-RADS v2.1	69	1	0.558	0.786	0.558	0.959	0.86
Sun et al. 2023 [11]	Biopsy	Proprietary Modular AI	PI-RADS v2.1	480	0.590	0.401	0.717	0.577	--	--
Giganti et al. 2025 [12]	Biopsy	DL-CAD (Lucida Pi v2.4)	PI-RADS v2.1	252	0.950	0.990	0.670	0.730	0.910	0.95
Bayerl et al. (Park) 2024 [13]	Biopsy	mdprostate (3D U-Net)	PI-RADS v2.1	1406	0.855	0.810	0.630	0.820	0.803	0.89
Bayerl et al. (Oerther) 2024 [13]	Biopsy	mdprostate (3D U-Net)	PI-RADS v2.1	11,686	0.855	0.890	0.630	0.660	0.803	0.89
Cai et al. (Internal) 2024 [14]	Biopsy	3D CNN (Image-only)	PI-RADS v2.1	400	0.790	0.850	0.840	0.870	0.890	0.89
Cai et al. (External) 2024 [14]	Biopsy	3D CNN (Image-only)	PI-RADS v2.1	204	0.830	0.870	0.730	0.750	0.860	0.84
Schrader et al. 2024 [15]	Biopsy	nnUNet Ensemble	PI-RADS v2.1	517	0.920	0.940	0.570	0.560	0.890	0.89
Youn et al. 2021 [16]	Biopsy/prostatectomy	Prostate AI v1.2.1	PI-RADS v2.1	121	0.767	0.930	0.859	0.780	0.828	0.914
Yu et al. (Internal) 2023 [17]	Biopsy	PI-RADSAI (UNet-Seg + ResNet)	PI-RADS v2.1	322	0.944	0.851	0.339	0.567	0.801	0.77
Yu et al. (External) 2023 [17]	Biopsy	PI-RADSAI (UNet-Seg + ResNet)	PI-RADS v2.1	153	0.827	0.867	0.618	0.691	0.833	0.867
Zhao et al. (SKH) 2023 [18]	Biopsy/prostatectomy	ResNet3D (DL-CS-Res)	PI-RADS v2.1	97	1.000	1.000	0.320	0.311	0.848	0.86
Zhao et al. (TZH) 2023 [18]	Biopsy/prostatectomy	ResNet3D (DL-CS-Res)	PI-RADS v2.1	248	0.992	0.917	0.453	0.477	0.885	0.860
Zhao et al. (CSH + SQH) 2023 [18]	Biopsy/prostatectomy	ResNet3D (DL-CS-Res)	PI-RADS v2.1	295	0.986	1.000	0.435	0.435	0.852	0.863

PI-RADS: Prostate Imaging Reporting and Data System; AUROC: Area under the Receiver Operating Characteristic curve; Rad: Radiologists. The two studies by Pellicer-Valero are based on different datasets: IVO refers to clinical data from the Instituto Valenciano de Oncología (Valencian Institute of Oncology), while ProstateX is a publicly available dataset from a prostate MRI challenge. In Bayerl et al.’s work, “Park” and “Oerther” denote subgroups led by different radiologists to reflect inter-reader variability. “Internal” and “External” in Cai et al.’s studies represent internal hospital data and external validation cohorts, respectively, used to assess generalizability. The three subgroups in Zhao et al.’s work—SKH, TZH, and CSH + SQH—refer to data collected from different hospitals: Shenzhen Second People’s Hospital (SKH), Shenzhen Cancer Hospital (TZH), and a combined cohort from The Sixth Affiliated Hospital of Sun Yat-sen University and Shunde First People’s Hospital (CSH + SQH).

## Data Availability

The original contributions presented in this study are included in the article. Further inquiries can be directed to the corresponding authors.

## References

[1] Siegel R.L., Kratzer T.B., Giaquinto A.N., Sung H., Jemal A. (2025). Cancer statistics, 2025. CA Cancer J. Clin..

[2] Cornford P., van den Bergh R.C.N., Briers E., Van den Broeck T., Brunckhorst O., Darraugh J., Eberli D., De Meerleer G., De Santis M., Farolfi A. (2024). EAU-EANM-ESTRO-ESUR-ISUP-SIOG Guidelines on Prostate Cancer-2024 Update. Part I: Screening, Diagnosis, and Local Treatment with Curative Intent. Eur. Urol..

[3] O’Shea A., Harisinghani M. (2022). PI-RADS: Multiparametric MRI in prostate cancer. Magn. Reson. Mater. Phys. Biol. Med..

[4] Padhani A.R., Godtman R.A., Schoots I.G. (2024). Key learning on the promise and limitations of MRI in prostate cancer screening. Eur. Radiol..

[5] Gassenmaier S., Afat S., Nickel D., Mostapha M., Herrmann J., Othman A.E. (2021). Deep learning-accelerated T2-weighted imaging of the prostate: Reduction of acquisition time and improvement of image quality. Eur. J. Radiol..

[6] Syer T., Mehta P., Antonelli M., Mallett S., Atkinson D., Ourselin S., Punwani S. (2021). Artificial Intelligence Compared to Radiologists for the Initial Diagnosis of Prostate Cancer on Magnetic Resonance Imaging: A Systematic Review and Recommendations for Future Studies. Cancers.

[7] Roest C., Fransen S.J., Kwee T.C., Yakar D. (2022). Comparative Performance of Deep Learning and Radiologists for the Diagnosis and Localization of Clinically Significant Prostate Cancer at MRI: A Systematic Review. Life.

[8] Page M.J., McKenzie J.E., Bossuyt P.M., Boutron I., Hoffmann T.C., Mulrow C.D., Shamseer L., Tetzlaff J.M., Akl E.A., Brennan S.E. (2021). The PRISMA 2020 statement: An updated guideline for reporting systematic reviews. BMJ.

[9] Saha A., Bosma J.S., Twilt J.J., van Ginneken B., Bjartell A., Padhani A.R., Bonekamp D., Villeirs G., Salomon G., Giannarini G. (2024). Artificial intelligence and radiologists in prostate cancer detection on MRI (PI-CAI): An international, paired, non-inferiority, confirmatory study. Lancet Oncol..

[10] Pellicer-Valero O.J., Marenco Jiménez J.L., Gonzalez-Perez V., Casanova Ramón-Borja J.L., Martín García I., Barrios Benito M., Pelechano Gómez P., Rubio-Briones J., Rupérez M.J., Martín-Guerrero J.D. (2022). Deep learning for fully automatic detection, segmentation, and Gleason grade estimation of prostate cancer in multiparametric magnetic resonance images. Sci. Rep..

[11] Sun Z., Wang K., Kong Z., Xing Z., Chen Y., Luo N., Yu Y., Song B., Wu P., Wang X. (2023). A multicenter study of artificial intelligence-aided software for detecting visible clinically significant prostate cancer on mpMRI. Insights Imaging.

[12] Giganti F., Moreira da Silva N., Yeung M., Davies L., Frary A., Ferrer Rodriguez M., Sushentsev N., Ashley N., Andreou A., Bradley A. (2025). AI-powered prostate cancer detection: A multi-centre, multi-scanner validation study. Eur. Radiol..

[13] Bayerl N., Adams L.C., Cavallaro A., Bäuerle T., Schlicht M., Wullich B., Hartmann A., Uder M., Ellmann S. (2024). Assessment of a fully-automated diagnostic AI software in prostate MRI: Clinical evaluation and histopathological correlation. Eur. J. Radiol..

[14] Cai J.C., Nakai H., Kuanar S., Froemming A.T., Bolan C.W., Kawashima A., Takahashi H., Mynderse L.A., Dora C.D., Humphreys M.R. (2024). Fully Automated Deep Learning Model to Detect Clinically Significant Prostate Cancer at MRI. Radiology.

[15] Schrader A., Netzer N., Hielscher T., Görtz M., Zhang K.S., Schütz V., Stenzinger A., Hohenfellner M., Schlemmer H.P., Bonekamp D. (2024). Prostate cancer risk assessment and avoidance of prostate biopsies using fully automatic deep learning in prostate MRI: Comparison to PI-RADS and integration with clinical data in nomograms. Eur. Radiol..

[16] Youn S.Y., Choi M.H., Kim D.H., Lee Y.J., Huisman H., Johnson E., Penzkofer T., Shabunin I., Winkel D.J., Xing P. (2021). Detection and PI-RADS classification of focal lesions in prostate MRI: Performance comparison between a deep learning-based algorithm (DLA) and radiologists with various levels of experience. Eur. J. Radiol..

[17] Yu R., Jiang K.W., Bao J., Hou Y., Yi Y., Wu D., Song Y., Hu C.H., Yang G., Zhang Y.D. (2023). PI-RADS(AI): Introducing a new human-in-the-loop AI model for prostate cancer diagnosis based on MRI. Br. J. Cancer.

[18] Zhao L., Bao J., Qiao X., Jin P., Ji Y., Li Z., Zhang J., Su Y., Ji L., Shen J. (2023). Predicting clinically significant prostate cancer with a deep learning approach: A multicentre retrospective study. Eur. J. Nucl. Med. Mol. Imaging.

[19] McGuinness L.A., Higgins J.P.T. (2021). Risk-of-bias VISualization (robvis): An R package and Shiny web app for visualizing risk-of-bias assessments. Res. Synth. Methods.

[20] DerSimonian R., Laird N. (1986). Meta-analysis in clinical trials. Control Clin. Trials.

[21] Hartung J., Knapp G. (2001). On tests of the overall treatment effect in meta-analysis with normally distributed responses. Stat. Med..

[22] Higgins J.P.T., Thompson S.G., Deeks J.J., Altman D.G. (2003). Measuring inconsistency in meta-analyses. BMJ.

[23] DeLong E.R., DeLong D.M., Clarke-Pearson D.L. (1988). Comparing the areas under two or more correlated receiver operating characteristic curves: A nonparametric approach. Biometrics.

[24] Borenstein M., Hedges L.V., Higgins J.P.T., Rothstein H.R. (2010). A basic introduction to fixed-effect and random-effects models for meta-analysis. Res. Synth. Methods.

[25] Egger M., Davey Smith G., Schneider M., Minder C. (1997). Bias in meta-analysis detected by a simple, graphical test. BMJ.

[26] Sun Z., Wang K., Gao G., Wang H., Wu P., Li J., Zhang X., Wang X. (2025). Assessing the Performance of Artificial Intelligence Assistance for Prostate MRI: A Two-Center Study Involving Radiologists With Different Experience Levels. J. Magn. Reson. Imaging.

[27] Wang K., Xing Z., Kong Z., Yu Y., Chen Y., Zhao X., Song B., Wang X., Wu P., Wang X. (2023). Artificial intelligence as diagnostic aiding tool in cases of Prostate Imaging Reporting and Data System category 3: The results of retrospective multi-center cohort study. Abdom. Radiol..

[28] Simon G.J., Aliferis C. (2024). Artificial Intelligence and Machine Learning in Health Care and Medical Sciences: Best Practices and Pitfalls.

[29] Gillies R.J., Kinahan P.E., Hricak H. (2016). Radiomics: Images Are More than Pictures, They Are Data. Radiology.

